# Ultrasound-Assisted Extraction of Yellow Peacock Flower (*Caesalpinia pulcherrima*) and Its Application in Gelatin Capsule Waste-Based Active Packaging Films for Dried Shrimp Preservation

**DOI:** 10.3390/antiox15050576

**Published:** 2026-05-02

**Authors:** Pudthaya Kumnerdsiri, Khanittha Chinarak, Lalitphan Kitsanayanyong, Anurak Uchuwittayakul, Wanchat Sirisarn, Piyangkun Lueangjaroenkit, Pimonpan Kaewprachu, Jaksuma Pongsetkul, Samart Saiut, Saroat Rawdkuen, Passakorn Kingwascharapong

**Affiliations:** 1 Department of Fishery Products, Faculty of Fisheries, Kasetsart University, Bangkok 10900, Thailand; pudthaya.ku@ku.th (P.K.); khanittha_noo@hotmail.com (K.C.); ffislhk@ku.ac.th (L.K.); 2 Department of Aquaculture, Faculty of Fisheries, Kasetsart University, Bangkok 10900, Thailand; ffisarb@ku.ac.th; 3 Department of Microbiology, Faculty of Medicine, Kasetsart University, Bangkok 10900, Thailand; wanchat.s@ku.th; 4 Department of Microbiology, Faculty of Science, Kasetsart University, Bangkok 10900, Thailand; piyangkun.lu@ku.th; 5 Faculty of Agro-Industry, Chiang Mai University, Samut Sakhon 74000, Thailand; pimonpan.k@cmu.ac.th; 6 School of Animal Technology and Innovation, Institute of Agricultural Technology, Suranaree University of Technology, Nakhon Ratchasima 30000, Thailand; jaksuma@sut.ac.th; 7 Department of Food Science, Faculty of Science, Burapha University, Chonburi 20131, Thailand; samarts@go.buu.ac.th; 8 Unit of Innovative Food Packaging and Biomaterials, School of Agro-Industry, Mae Fah Luang University, Chiang Rai 57100, Thailand; saroat@mfu.ac.th

**Keywords:** *Caesalpinia pulcherrima*, gelatin capsule waste, biodegradable films, ultrasound-assisted extraction, antioxidant activity

## Abstract

Environmental pollution from plastics is largely driven by inadequate waste management, particularly in food packaging that relies heavily on petroleum-derived materials. This study utilized gelatin capsule waste (GCW) as a sustainable biopolymer and incorporated yellow peacock flower extract (YPE), obtained via ultrasound-assisted extraction (UAE), at various concentrations (0–2%, *w*/*v*) to develop biodegradable films with enhanced functional and antioxidant properties. The main phenolic constituents of YPE were flavonoid aglycones and their glycosylated derivatives. YPE showed total phenolic content of 98.44–129.34 mg GAE/g dry extract, with ABTS, DPPH, and FRAP antioxidant activities ranging from 5.51 to 8.11, 3.17–7.63, and 3.86–5.82 mg TE/g dry extract, respectively. Incorporation of YPE into GCW films significantly improved light barrier properties, thermal stability, mechanical strength, and antioxidant activity, along with a reduction in water vapor permeability and an increase in contact angle, indicating enhanced film hydrophobicity. All films exhibited excellent biodegradability, with complete disintegration within 15 days under soil burial conditions. Films containing 2% YPE (GF4) showed significantly higher thickness, tensile strength, and thermal stability, along with increased opacity, compared with the control (GF0), indicating a reinforcing effect. FTIR analysis revealed the interaction between protein and phenolic compounds from YPE. In a food application model, GF4 film pouches (5 × 5 cm^2^) effectively delayed oxidative deterioration of dried shrimp during storage at 25 ± 2 °C for 15 days. These findings highlight YPE as a promising bioactive ingredient for biodegradable active packaging and demonstrate the feasibility of GCW as a sustainable biopolymer for eco-friendly films.

## 1. Introduction

Petroleum-based plastics have long dominated the packaging industry due to their low production costs, favorable physicochemical properties, and practical convenience [1,2]. However, the persistent and non-biodegradable nature of these plastics, together with improper disposal and inefficient post-consumer waste management, has led to their accumulation in the environment, driving the growing demand for sustainable alternatives. Consequently, the development of biodegradable packaging materials has attracted considerable attention as an effective strategy to mitigate the ecological impact of conventional plastics [3,4,5]. Numerous biopolymers derived from renewable plant and animal sources, including proteins, polysaccharides, and lipids, have been investigated for their potential as environmentally friendly alternatives [6]. Among the various biopolymers, gelatin has emerged as a promising candidate for food packaging applications due to its low cost, abundance, renewability, biodegradability, and edibility [7]. Gelatin is a natural protein obtained from the partial hydrolysis of collagen, typically derived from animal by-products such as the bones and skin of fish, pigs, cows, camels, goats, and jellyfish [7,8]. However, the inadequate barrier and mechanical properties of natural biopolymer films remain a major limitation for their application in food packaging [9]. To overcome these limitations, natural biopolymers have been blended with synthetic polymers or enriched with natural extracts to enhance the functional properties of the resulting materials [10].

In the pharmaceutical sector, the production of hard gelatin capsules generates a substantial amount of solid waste, estimated at approximately 1000 kg per month according to industrial reports from a capsule manufacturing facility in Pathum Thani, Thailand [11,12]. This waste is generally unsuitable for direct reuse, as the high-temperature and chemical processing steps involved in capsule production lead to the degradation of key functional properties, including light and water barrier performance as well as solubility [13]. Utilizing this waste is essential, as it provides a sustainable strategy to reduce disposal costs, minimize environmental pollution, and enhance resource efficiency [14]. Developing biodegradable packaging from gelatin capsule waste offers a promising valorization pathway, enabling the conversion of underutilized by-products into functional materials with added environmental value.

Edible flowers are valuable sources of phytochemicals, especially phenolic compounds, which impart strong antioxidant properties and associated health benefits [15]. *Caesalpinia pulcherrima* L. (yellow peacock) is an edible flower commonly used in Thai cuisine and teas and is recognized for its high phenolic content, which imparts strong antioxidant activity and nutraceutical value [15,16], making it a promising active ingredient. However, the recovery of bioactive compounds from edible flower extracts depends on several factors, including the extraction method and extraction conditions.

Ultrasound-assisted extraction (UAE) is an efficient green extraction technique that enables higher recovery of natural compounds while reducing solvent consumption and extraction time [17]. Owing to its high extraction efficiency and environmental sustainability, UAE has emerged as a promising approach for the recovery of bioactive compounds from plant- and flower-derived matrices [18]. Despite the growing interest in ultrasound-assisted extraction for recovering bioactive compounds from edible flowers, studies focusing on the extraction of yellow peacock flower extract (YPE), its incorporation into gelatin capsule waste–based films, and their application in food model systems remain limited. Accordingly, this study aimed to (i) evaluate the effects of UAE conditions on the antioxidant and phenolic compounds profile of YPE; (ii) investigate the influence of YPE at different concentrations on the mechanical, thermal, and antioxidant properties of gelatin capsule waste (GCW)-based films; and (iii) assess the quality of dried shrimp packaged in pouches made from the developed films during storage.

## 2. Materials and Methods

### 2.1. Sample Collection

*Caesalpinia pulcherrima* (L.) flowers were collected from natural vegetation in Bangkok, Thailand, in June 2025. The flowers were collected fresh and transported to the laboratory for further processing. Gelatin capsule waste (GCW) was obtained from Service Pack Manufacturing Company Ltd., Pathum Thani, Thailand. All chemicals used in this study were of analytical grade.

### 2.2. Preparation of Yellow Peacock Extract (YPE) Using Ultrasound-Assisted Extraction

Yellow peacock flowers were rinsed with distilled water and dried in a hot-air oven at 60 °C for 24 h. The dried flowers were ground using a blender and passed through an 80-mesh stainless steel sieve to obtain a fine powder. The powdered sample was mixed with distilled water at a ratio of 1:10 (*w*/*v*). A maceration method was used as the control treatment and designated as E0. For ultrasound-assisted extraction (UAE), the sample was processed using a Vibra-Cell ultrasonic processor (Sonic & Materials, Inc., Newtown, CT, USA) at two amplitude levels (20% and 40%) and three extraction times (15, 30, and 45 min). The treatments were designated as follows: amplitude 20% for 15 min (E1), amplitude 20% for 30 min (E2), amplitude 20% for 45 min (E3), amplitude 40% for 15 min (E4), amplitude 40% for 30 min (E5), and amplitude 40% for 45 min (E6). The resulting extract was filtered through Whatman No. 1 filter paper and centrifuged at 5000× *g* for 30 min at 4 °C. The supernatant was collected and freeze-dried using a Labconco FreeZone 4.5 L benchtop freeze dryer (Kansas City, MO, USA) to obtain the Yellow Peacock Extract (YPE) in powdered form.

### 2.3. Preparation of Gelatin Capsule Waste (GCW) Films Incorporated with Yellow Peacock Extract (YPE)

The film-forming solution (FFS) was prepared following the method of Kumnerdsiri et al. [14]. Briefly, 50 g of gelatin capsule waste (GCW) was dissolved in 100 mL of distilled water and stirred at 60 °C for 30 min. Yellow Peacock Extract (YPE) was incorporated into the GCW solution at different concentrations (0.25, 0.5, 1.0, and 2.0% *w*/*v*), followed by continuous stirring for an additional 30 min to ensure complete homogenization. The resulting FFS was sonicated in an ultrasonic bath for 5 min to remove entrapped air bubbles. Afterwards, 10 g of the solution was cast into Petri dishes and dried at 35 °C for 24 h. The dried films were carefully peeled off and conditioned at room temperature prior to further characterization.

### 2.4. Analyses

#### 2.4.1. Total Phenolic Content (TPC)

The total phenolic content (TPC) of the YPE was determined using a 96-well microplate assay, following the method of Kumnerdsiri et al. [1] with minor modifications. Briefly, 100 µL of the extract sample was mixed with 100 µL of Folin–Ciocalteu reagent, followed by the addition of 300 µL of 7.5% (*w*/*v*) sodium carbonate solution. The mixture was incubated at room temperature for 30 min, and the absorbance was measured at 765 nm using a microplate reader. Gallic acid (Merck, Darmstadt, Germany) was used to construct the calibration curve. TPC values were expressed as milligrams of gallic acid equivalents per gram of dry extract (mg GAE/g dry extract).

#### 2.4.2. Antioxidant Activities of Yellow Peacock Flower Extract

The DPPH radical scavenging activity of the YPE was determined using a 96-well microplate assay, following the method of Kumnerdsiri et al. [1]. Briefly, 200 μL of the extract solution was mixed with 800 μL of 200 µM DPPH solution prepared in 85% ethanol and incubated at room temperature for 30 min. The absorbance of 200 μL aliquots was measured at 517 nm. A Trolox standard curve was constructed, and the results were expressed as milligrams Trolox equivalents per gram of dry extract (mg TE/g dry extract).

The ABTS radical scavenging activity of the YPE was determined using a 96-well microplate assay, according to the method of Kumnerdsiri et al. [1], with slight modifications. The ABTS•^+^ working solution was prepared by reacting 14.8 mM ABTS with 5.2 mM potassium persulfate, followed by incubation in the dark at room temperature for 16–18 h. The solution was then diluted with distilled water to obtain an absorbance of 0.700 ± 0.002 at 734 nm. For the assay, 25 µL of the extract solution was mixed with 975 µL of the diluted ABTS•^+^ and incubated at room temperature for 15 min. Absorbance was recorded at 734 nm using a microplate reader. A Trolox calibration curve was prepared, and the results were expressed as milligrams Trolox equivalents per gram of dry extract (mg TE/g dry extract).

The FRAP assay was conducted following the method of Kumnerdsiri et al. [1], with slight modifications. The FRAP reagent was prepared by mixing 10 mL of 300 mM acetate buffer (pH 3.6) with 1 mL of 10 mM TPTZ and 1 mL of 20 mM FeCl_3_ in a 10:1:1 ratio. Reactions were conducted in a 96-well plate by combining 450 μL of FRAP reagent with 50 μL of extract. After incubation for 30 min, absorbance was measured at 593 nm. The antioxidant reducing capacity was quantified and expressed as milligrams of Trolox equivalents per gram of dry extract (mg TE/g dry extract).

#### 2.4.3. LC-MS/MS Profiling and Identification of Bioactive Compounds

The evaluation of bioactive compounds in yellow peacock extract was conducted according to the method described by Kumnerdsiri et al. [1]. The extract was filtered through a 0.22 μm nylon syringe filter prior to LC-QTOF-MS/MS analysis. The filtered extract was analyzed using an LC-MS/MS-QTOF system (Sciex, ExionLC™ AD system; X500R QTOF system, Framingham, MA, USA). An aliquot of 20 µL was injected into a Kinetex^®^ XB-C18 column (1.7 µm, 100 × 2.1 mm, 2.6 µm particle size, 100 Å; Phenomenex, Torrance, CA, USA). The mobile phase consisted of solvent A (0.1% formic acid in water) and solvent B (0.1% formic acid in acetonitrile), delivered under isocratic conditions (50:50, *v*/*v*) at a flow rate of 0.5 mL/min. The LC-QTOF-MS/MS was operated in negative electrospray ionization (ESI) mode. The operating parameters were set as follows: spray voltage, 4500 V; curtain gas, 30 psi; ion source gas 1, 60 psi; gas 2, 70 psi; ion source temperature, 400 °C; scan range, *m*/*z* 50–1000 Da; declustering potential, 60 V; collision energy, 10 eV. Compound identification was carried out based on accurate mass measurement and MS/MS fragmentation patterns, using SCIEX OS software (version 4.0.2) with the AutoPeak algorithm and by comparison with the Natural Products HR-MS/MS Library 2.0 and previously reported literature. A mass error tolerance of ≤5 ppm was applied for compound assignment. Due to the lack of authentic reference standards, the identified compounds were considered as putatively annotated with a high level of confidence based on MS/MS spectral matching.

### 2.5. Film Analyses

#### 2.5.1. Color

The color attributes of gelatin capsule waste films incorporated with yellow peacock extract were measured using a CIE colorimeter (ColorFlex, HunterLab, Reston, VA, USA), assessing L* (lightness), a* (red/green), and b* (yellow/blue) values, and the color difference (ΔE) was manually calculated using the standard Equation (1).

(1) $$∆E=\sqrt{{\left({L*}_{sample}-{L*}_{control}\right)}^{2}+{\left({a*}_{sample}-{a*}_{control}\right)}^{2}+{\left({b*}_{sample}-{b*}_{control}\right)}^{2}}$$

#### 2.5.2. Thickness

The thickness of gelatin capsule waste films incorporated with yellow peacock extract was measured using a digital micrometer (Mitutoyo, Model ID-C112PM, Serial No. 00320, Mitutoyo Corp., Kawasaki-shi, Japan). Measurements were taken at five randomly selected points on each film sample, and the average thickness was calculated.

#### 2.5.3. Mechanical Properties

Mechanical properties, including tensile strength (TS) and elongation at break (EAB), were evaluated using a texture analyzer (Model TA.XT2i, Stable Micro Systems, Godalming, UK) according to the method described by Kumnerdsiri et al. [14,19].

#### 2.5.4. Light Transmittance and Transparency Values

The light transmittance and transparency of gelatin capsule waste films incorporated with yellow peacock extract were measured across the 200–800 nm wavelength range using a UV-Vis spectrometer (Shimadzu, Model UV-1900, Kyoto, Japan), following the method of Kumnerdsiri et al. [14]. Additionally, the transparency value was specifically measured at 600 nm and calculated according to Equation (2).

(2) $$\mathrm{Transparency}\;\mathrm{value}=\frac{-\log T600}{x}$$

T_600_ denotes the light transmitted at 600 nm, while x represents the film thickness (mm). The lower transparency value indicated that the film was more transparent [20].

#### 2.5.5. Moisture Content and Solubility

Film samples (2 cm × 2 cm) were weighed (W_0_) and dried in a hot air oven (Model WTB.1, Memmert, Schwabach, Germany) at 105 °C for 24 h. The dried films were then reweighed (W_1_), and moisture content (MC) was calculated using Equation (3).

(3) $$\mathrm{MC}\;(\%)=\frac{W_{0\;}-{\;W}_{1}}{W_{0}}\times \;100$$

The solubility of gelatin capsule waste films incorporated with yellow peacock extract was determined by measuring the percentage of dry matter dissolved after 24 h. of immersion in distilled water at room temperature. Dry film samples (2 cm × 2 cm) were weighed (W_i_) and submerged in 30 mL of distilled water, followed by vortex agitation. After 24 h, the mixture was filtered through filter paper to collect the retentate, which was then dried at 105 °C for 24 h. The dried insoluble residue was weighed (W_f_), and solubility was calculated using Equation (4).

(4) $$\mathrm{WS}\;(\%)=\frac{W_{i}\;-\;W_{f}}{W_{i}}\times \;100$$

#### 2.5.6. Contact Angle Measurement

Selected films were evaluated for hydrophobicity by measuring the water contact angle using the sessile drop technique with a commercial contact angle meter (DataPhysics Model OCA 15EC, Filderstadt, Germany) equipped with image analysis software, following the procedure of Tagrida et al. [21]. Film samples (5 cm × 5 cm) were placed on a movable, horizontally leveled stage. A 3 μL droplet of deionized water was dispensed onto the film surface using a 20 μL micro-injector. Contact angles were measured on both sides of the droplet, with five replicates taken at different locations on each film. All measurements were conducted under controlled conditions of 50 ± 5% relative humidity and 25 ± 0.5 °C.

#### 2.5.7. Water Vapor Permeability

The water vapor permeability (WVP) of the films was determined following the method of Tagrida et al. [21]. Film samples were mounted on aluminum permeation cups containing silica gel (0% RH). Silicone vacuum grease and rubber gaskets were used to seal the films tightly onto the cups. The cups were placed in a controlled environment chamber set at 25 ± 2 °C and 50 ± 5% RH and weighed at 1 h intervals over a 10 h period. WVP was calculated according to Equation (5).

(5) $$\mathrm{WVP}\;(g\;m^{-1}\;s^{-1}\;{\mathrm{Pa}}^{-1})=\frac{\mathrm{WVTR}\;\times \;L}{\Delta P\;\times \;A}$$

where WVTR is the water vapor transmission rate, determined from the slope of the plot of cup weight gain versus time (g s^−1^); L is the film thickness (m); ΔP is the water vapor pressure difference across the film (1583.74 Pa); and A is the exposed film area (m^2^).

#### 2.5.8. FTIR Spectroscopy

Film samples were analyzed using a Fourier-transform infrared (FTIR) spectrometer (Model 400, Perkin Elmer, Beaconsfield, UK) equipped with attenuated total reflectance (ATR) mode. Each film was placed on the ATR crystal and clamped securely. Spectra were recorded over the range of 400–4000 cm^−1^, with 64 scans accumulated at a resolution of 4 cm^−1^.

#### 2.5.9. Thermogravimetric Analysis (TGA)

Thermal stability of the films was assessed using thermogravimetric analysis (TGA) with a TGA 2 STARe system (Mettler Toledo, Greifensee, Switzerland). Film samples (10–15 mg) were placed in sample pans and heated from 25 °C to 800 °C at a rate of 10 °C/min under a nitrogen atmosphere (20 mL/min) to prevent thermo-oxidative degradation. Thermogravimetric curves were analyzed to determine the thermal degradation profiles, expressed as percentage weight loss of the films.

#### 2.5.10. Differential Scanning Calorimetry (DSC)

Differential scanning calorimetry (DSC) thermograms were recorded using a DSC 1 STARe System (Mettler Toledo, Greifensee, Switzerland). Film samples (1–2 mg) were weighed and sealed in aluminum pans. Samples were initially heated from 25 °C to 80 °C at a rate of 10 °C/min to remove moisture, cooled to −80 °C at the same rate, and then reheated to 300 °C at 10 °C/min. All measurements were performed under a nitrogen atmosphere with a flow rate of 50 mL/min. An empty aluminum pan served as the reference.

#### 2.5.11. Antioxidant Activities of Film 

Film samples (25 mg) were dissolved in 3 mL of distilled water and stirred to obtain a homogeneous solution. The antioxidant activities of the solutions were then evaluated using DPPH, ABTS, and FRAP assays, as described in Section 2.4.2.

#### 2.5.12. Biodegradability: Indoor Soil Degradation

Biodegradability was evaluated following the method of Kumnerdsiri et al. [14]. Film samples (2 cm × 3 cm) were weighed (W_0_), placed in plastic boxes, and buried in organic soil at room temperature for 15 days, with water added every 2 days to maintain moisture. After 15 days, the remaining films were retrieved and reweighed (W_T_). Biodegradability was expressed as weight loss (W_L_) and calculated using Equation (6).

(6) $$W_{L}\;(\%)=\frac{W_{0}\;-\;W_{T}}{W_{0}}\times \;100$$

### 2.6. Packaging Procedure and Storage Conditions

The formulated films were fabricated into pouches measuring 5 × 5 cm. Fresh dried shrimp (1 g) was placed into each pouch prepared from the different film formulations. The air inside the pouches was manually removed before sealing with a heat sealer. For comparison, a positive-control group was prepared using commercially available polyethylene (PE) Zip-lock pouches, representing conventional packaging material. All packaged samples were stored at ambient temperature (25 ± 2 °C). During the storage period, samples were randomly collected on days 0, 3, 7, 10, and 14 for subsequent quality analyses, including visual appearance, color, and Thio-Barbituric Acid Reactive Substance (TBARS).

#### 2.6.1. Visual Appearances and Color

The visual appearance of dried shrimp samples was evaluated for surface uniformity and discoloration by visual observation under natural daylight. Representative photographs were taken at each storage interval to document visible changes in color.

The color attributes were further quantified using a CIE colorimeter (ColorFlex, HunterLab, Reston, VA, USA). The parameters L* (lightness), a* (redness/greenness), and b* (yellowness/blueness) were measured at three random positions on the shrimp surface. All measurements were performed in triplicate, and the average values were used to represent the color characteristics of each treatment.

#### 2.6.2. Moisture Content (MC)

The moisture content (MC) of the dried shrimp samples was determined gravimetrically. Briefly, 0.5 g of sample was accurately weighed (W_0_) and dried in a hot air oven (Model WTB.1, Memmert, Schwabach, Germany) at 105 °C for 24 h until a constant weight was obtained. The dried samples were then reweighed (W_1_), and the MC was calculated using Equation (2) (as described in Section 2.5.5).

#### 2.6.3. Thio-Barbituric Acid Reactive Substances (TBARS)

The lipid oxidation of the dried shrimp samples was evaluated by determining the thio-barbituric acid reactive substances (TBARS) value according to the method of Theerawitayaart et al. [22], with slight modifications. Briefly, 0.05 g of dried shrimp was mixed with 2.5 mL of thiobarbituric acid (TBA) reagent containing 0.375 g of TBA, 15 g of trichloroacetic acid (TCA), and 0.875 mL of hydrochloric acid (HCl) in 100 mL of distilled water. The mixture was heated in a boiling water bath (95–100 °C) for 10 min to develop a pink-colored chromogen. After heating, the samples were immediately cooled under running tap water and centrifuged at 7500× *g* for 10 min. The absorbance of the supernatant was measured at 532 nm using a UV–Vis spectrophotometer. The TBARS value was determined from a standard curve of malondialdehyde (MDA) and expressed as mg MDA/kg dried sample.

### 2.7. Statistical Analysis

A completely randomized design (CRD) was employed in this study. Data is presented as means ± standard deviation (SD). Statistical analyses were conducted using analysis of variance (ANOVA), followed by Duncan’s multiple range test to determine significant differences between treatments. All analyses were performed using SPSS software (Version 23.0, SPSS Inc., Chicago, IL, USA).

## 3. Results and Discussion

### 3.1. Total Phenolic Content

The total phenolic content (TPC) of YPE is shown in Table 1. The control sample (E0), obtained by maceration extraction, showed the lowest TPC (88.14 mg GAE/g dry extract) compared with the other samples, whereas higher TPC values were observed in YPE obtained using UAE, regardless of extraction level. This enhancement can be attributed to intensified acoustic cavitation during UAE, where the rapid adiabatic compression and collapse of microbubbles generate localized hotspots with extreme temperature and pressure, leading to cell wall disruption and enhanced release of phenolic compounds into the extraction medium [23]. Increasing the ultrasonic amplitude from 20% to 40% led to a marked increase in TPC, from 102.94 to 129.34 mg GAE/g dry extract, with the highest value observed at 40% (E6). This effect is attributed to enhanced cavitation, which intensifies microjet impacts and shear forces, thereby promoting cell wall disruption and the release of bound phenolics [1]. Similarly, an increase in extraction time from 15 to 45 min led to a progressive rise in TPC. This improvement can be attributed to the prolonged exposure of plant tissues to ultrasonic cavitation, which enhances cell wall permeability and promotes the diffusion of phenolic compounds into the solvent phase [24]. These results are in agreement with the findings of Dip et al. [25], who observed that both increasing ultrasonic power and extending extraction time significantly enhanced the TPC of *Bauhinia variegata* flower extracts. This indicates that the efficiency of phytochemical recovery from floral matrices is strongly dependent on both sonication intensity and extraction duration.

### 3.2. Antioxidant Activities of Yellow Peacock Flower Extract

The antioxidant activities of YPE, determined through DPPH, ABTS, and FRAP assays, are presented in Table 1. Among all treatments, the extract obtained at 40% amplitude for 45 min (E6) exhibited the highest antioxidant capacity across all assays (DPPH = 7.63 ± 0.20, ABTS = 8.11 ± 0.01, and FRAP = 5.82 ± 0.22 mg TE/g dry extract). This improvement can be attributed to acoustic cavitation generated during ultrasound treatment, which enhances cell wall disruption and facilitates the release of phenolic constituents and other antioxidant compounds [1]. The synergistic effect of increased ultrasonic amplitude and prolonged extraction time enhances cavitation intensity and solvent penetration, leading to more efficient polyphenol liberation and superior antioxidant potential [26]. These results are in agreement with the observations of Um et al. [27], who reported a comparable enhancement in the antioxidant capacity of *Rosa rugosa* fruit extracts under intensified ultrasonic conditions. Accordingly, sample E6 was selected for the evaluation of phenolic compound profiles and for incorporation into the film formulation, as it exhibited the highest TPC and antioxidant activity among all treatments.

### 3.3. Identification of Phenolic Compounds in Yellow Peacock Flower Extract

Qualitative LC-MS/MS analysis was employed to compare the phenolic profiles of the YPE control extract (E0) and the extract obtained under optimized UAE conditions (E6) (Table 2). Nine compounds were identified in each extract. Notably, amber acid was exclusively detected in the E0, whereas bavachinin A was uniquely present in the E6 extract, indicating compositional differences influenced by the extraction conditions. LC–MS/MS analysis revealed a diverse range of phenolic-related compounds, including flavonoids (e.g., luteoloside and bavachinin), phenolic acids (e.g., caleorioside B, gallic acid, and quinic acid), and glycosides (e.g., resibufogenin) [28]. Notably, the detection of bavachinin A in the E6 extract may account for its superior radical scavenging capacity. Bavachinin, a flavonoid phytochemical, has been reported to exhibit multiple bioactivities, including antibacterial, antioxidant, anti-inflammatory, α-glucosidase inhibitory, and nitric oxide inhibitory effects [29,30]. This finding suggests that UAE may improve the extraction efficiency of phenolic compounds from YPE. Similarly, previous research of [31], who demonstrated that optimized UAE conditions improve phenolic compound extraction from senggani flowers, supporting its potential as a natural antioxidant source. In addition, the coexistence of flavonoid glycosides and low-molecular-weight phenolic acids is typically linked to hydrogen atom transfer (HAT)-based antioxidant mechanisms, which is consistent with the strong radical scavenging activities observed in the DPPH and ABTS assays, as reported by Upadhyay et al. [32]. A similar observation was reported by Raha et al. [33], who identified multiple phenolic constituents in the methanolic extract of *C. pulcherrima* leaves via LC–MS, most of which exhibited antioxidant activities. Therefore, the LC–MS/MS results suggest that the phenolic constituents in YPE, particularly those obtained under the optimized UAE condition (E6), collectively contribute to its pronounced antioxidant activity. These findings highlight the potential of yellow peacock flower extract as a natural preservative or an active ingredient in edible coatings and packaging systems.

### 3.4. Color and Appearance

**Figure 1:**
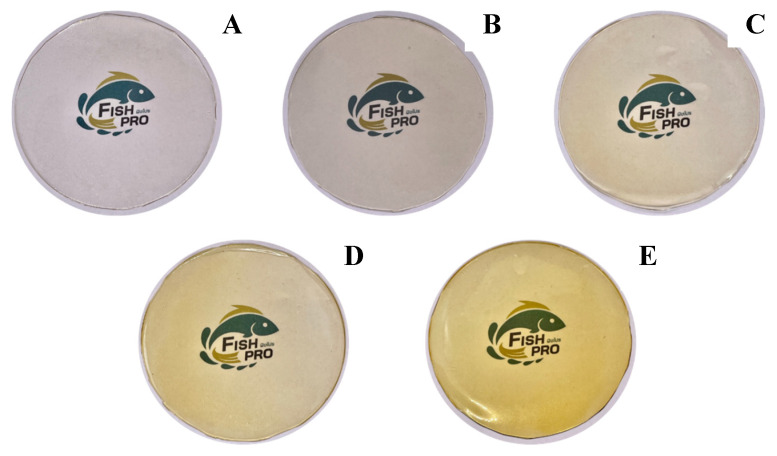
Photographs of gelatin capsule waste films incorporated with different levels of yellow peacock flower extract: (**A**) GF0, (**B**) GF1, (**C**) GF2, (**D**) GF3, and (**E**) GF4. GF0 represents gelatin film without yellow peacock extract, while GF1–GF4 represent films containing 0.25%, 0.5%, 1%, and 2% extract, respectively.

The color characteristics of gelatin capsule waste films containing different concentrations of YPE are summarized in Table 3 and visually illustrated in Figure 1. The control film (GF0) was colorless and exhibited the highest lightness (L* = 88.68). As the YPE concentration increased (GF1–GF4 ), the L* value significantly decreased to 77.74, while both a* and b* values increased (*p* < 0.05), indicating a progressive shift toward a more intense yellow coloration. This trend was consistent with the observed ΔE values. In general, ΔE values between 0.2 and 0.5 indicate slight color differences that are barely noticeable, whereas values above 6.0 represent clear and easily visible differences [34]. These visual changes were also clearly reflected in the film appearance (Figure 1). The intensified yellow coloration can be attributed to the phenolic constituents in YPE, which function as natural pigments [35]. A comparable trend was reported by Han Lyn et al. [36], who observed a decrease in L* and an increase in a* and b* values when *Clitoria ternatea* extract was incorporated into gelatin-based films. Overall, the addition of YPE markedly altered the color parameters of the gelatin capsule waste films, confirming its significant influence on film pigmentation.

### 3.5. Thickness

Film thickness is a key parameter that directly affects the physical and mechanical behaviors of biopolymeric films [37]. As presented in Table 4, the thickness of gelatin capsule waste films increased significantly (*p* < 0.05), ranging from 0.3018 to 0.3472 mm as the concentration of YPE increased, with GF4 exhibiting the highest thickness. This result can be attributed to interactions between gelatin and YPE, which led to a more compact arrangement within the film network [38,39]. A similar trend was observed by do Nascimento et al. [40], who demonstrated that increasing levels of *Clitoria ternatea* extract resulted in thicker starch–PVA films. Moreover, the interaction between gelatin and YPE may reduce the availability of hydrophilic groups for water binding, in agreement with the lower MC values observed in Table 4, similar to those reported by Nilsuwan et al. [41]. These results indicate that YPE concentration is a determining factor in modulating the thickness of gelatin capsule waste films.

### 3.6. Mechanical Properties

The mechanical properties of the gelatin capsule waste films, expressed as tensile strength (TS) and elongation at break (EAB), are presented in Table 4. The incorporation of YPE resulted in a significant increase in TS and a concomitant decrease in EAB (*p* < 0.05). Among all formulations, GF4 exhibited the highest TS (2.80 MPa) and the lowest EAB (13.67%), compared with the control film (GF0). This enhancement in mechanical strength can be attributed to the development of a more compact and cohesive polymeric matrix, likely driven by hydrogen bonding and other intermolecular interactions between gelatin chains and phenolic constituents of YPE [42]. FTIR analysis (the figure in Section 3.12) further confirmed these interactions through detectable shifts in characteristic functional groups. The reduction in EAB suggests restricted molecular mobility within the film network, a phenomenon commonly observed when polyphenol-protein interactions limit chain flexibility [43]. A similar trend was reported by Valizadeh et al. [44], who found that increasing pomegranate flower extract enhanced TS while reducing EAB in sage seed gum/gelatin films. Collectively, these results indicate that YPE acts as a natural reinforcing agent, improving the structural integrity of gelatin capsule waste films.

### 3.7. Light Transmittance and Transparency Value

**Figure 2:**
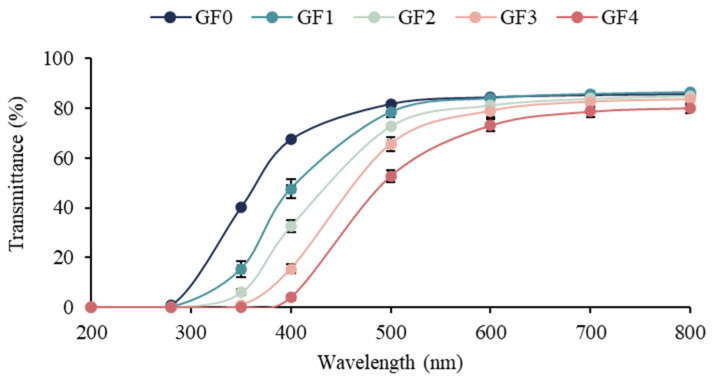
Light transmittance of gelatin capsule waste films incorporated with different levels of yellow peacock flower extract. GF0 represents gelatin film without yellow peacock extract, while GF1–GF4 represent films containing 0.25%, 0.5%, 1%, and 2% extract, respectively.

The light transmittance values of the gelatin capsule waste films containing different levels of YPE are shown in Table 4, and Figure 2. As expected, the control gelatin film (GF0) exhibited intrinsic UV-absorbing ability due to the presence of aromatic amino acids (e.g., tyrosine, phenylalanine, and tryptophan), which act as chromophoric groups and mainly absorb light below 300 nm [45]. The incorporation of YPE significantly enhanced the UV and visible light barrier properties of the films (*p* < 0.05). This improvement can be attributed to the high phenolic content of YPE, as phenolic compounds contain conjugated double bonds and aromatic rings capable of absorbing UV radiation. In addition, phenolic–gelatin interactions and the formation of a denser film matrix may increase light scattering, further reducing transmittance [46]. Similar trends were observed by Sun et al. [47], who reported enhanced UV shielding in gelatin/CMC-based films incorporated with coffee leaf extract. Therefore, the incorporation of YPE effectively improves the light barrier properties of gelatin capsule waste films, highlighting their potential as UV-protective materials for active food packaging.

Transparency plays a key role in consumer perception, especially for packaging applications in which visual inspection of the product is required [48]. As presented in Table 3 and Figure 1, the transparency value of the film significantly increased with the incorporation of YPE, indicating higher opacity compared to the control film (GF0) (*p* < 0.05). The higher transparency value indicates that the film is more opaque [20]. This trend is consistent with Fang et al. [49], who reported increased opacity in gelatin-chitosan films enriched with finger millet polyphenols. The increased in opacity is likely associated with the presence of phenolic compounds, which enhance light scattering and absorb visible and UV radiation, thereby reducing film clarity. Overall, the addition of YPE introduces a functional balance: while the films become more opaque, their UV-blocking ability improves, demonstrating a practical trade-off between visual clarity and protective performance.

### 3.8. Moisture Content

The moisture content of gelatin capsule waste films incorporating different concentrations of YPE is shown in Table 4. The control film (GF0) exhibited the highest moisture content (19.58%). In contrast, films containing 0.5–2% (*w*/*v*) YPE showed a significant decrease in moisture content (*p* < 0.05). This reduction can be attributed to the relatively hydrophobic and structurally rigid phenolic constituents in YPE, which may establish additional hydrogen bonding or hydrophobic interactions with gelatin amino acid residues, thereby restricting the availability of binding sites for water molecules [36,50]. A comparable trend was reported by Shakouri et al. [51], who observed reduced moisture content in gelatin–basil seed gum films upon the incorporation of saffron petal extract. Although increased YPE levels improve water resistance, excessive reduction in moisture content may lead to films that are more brittle, potentially limiting their functionality in high-moisture food systems.

### 3.9. Water Solubility

The water solubility of gelatin capsule waste films incorporated with different levels of YPE is shown in Table 4. Water solubility is a key indicator of film functionality, as it directly influences storage stability, handling properties, and suitability for specific applications. While low solubility enhances moisture resistance and structural integrity, higher solubility may be advantageous for edible or cookable films, as well as for promoting biodegradability [48]. The control film (GF0) exhibited the highest solubility (88.78%), which is expected due to the inherently hydrophilic nature of gelatin. In contrast, the incorporation of YPE significantly reduced film solubility (*p* < 0.05). This decrease can be attributed to the hydrophobic phenolic compounds in YPE, which may interact with gelatin chains through hydrogen bonding or hydrophobic interactions, thereby limiting water penetration and dissolution of the polymer network [52]. A similar reduction in solubility was reported by Musso et al. [53] in gelatin films containing red cabbage extract. Overall, YPE incorporation improves the water resistance of gelatin capsule waste films, suggesting its potential for applications requiring low-solubility, moisture-stable biopolymer films.

### 3.10. Water Vapor Permeability (WVP)

Water vapor permeability (WVP) represents the rate at which water vapor diffuses through a film and is a key parameter for evaluating the moisture barrier performance of biodegradable packaging materials [21]. The WVP values of gelatin capsule waste films incorporating different concentrations of YPE are summarized in Table 4. The control film (GF0) exhibited the highest WVP, whereas the film containing 2% YPE (GF4) showed the lowest value (*p* < 0.05), indicating a marked improvement in barrier efficiency. The reduction in WVP following YPE incorporation can be attributed to the presence of phenolic compounds, which may interact with the gelatin matrix through hydrogen bonding or hydrophobic interactions. These interactions likely decrease the number of available polar sites within the gelatin network, thereby reducing water adsorption and hindering vapor diffusion across the film structure [54]. Comparable results were reported by Kord et al. [55], who demonstrated that plant-derived phenolics improved the moisture barrier properties of fish gelatin films. Overall, the incorporation of YPE effectively enhances the water vapor barrier capacity of gelatin capsule waste films, reinforcing their potential suitability for moisture-sensitive food packaging applications.

### 3.11. Water Contact Angle (WCA)

**Figure 3:**
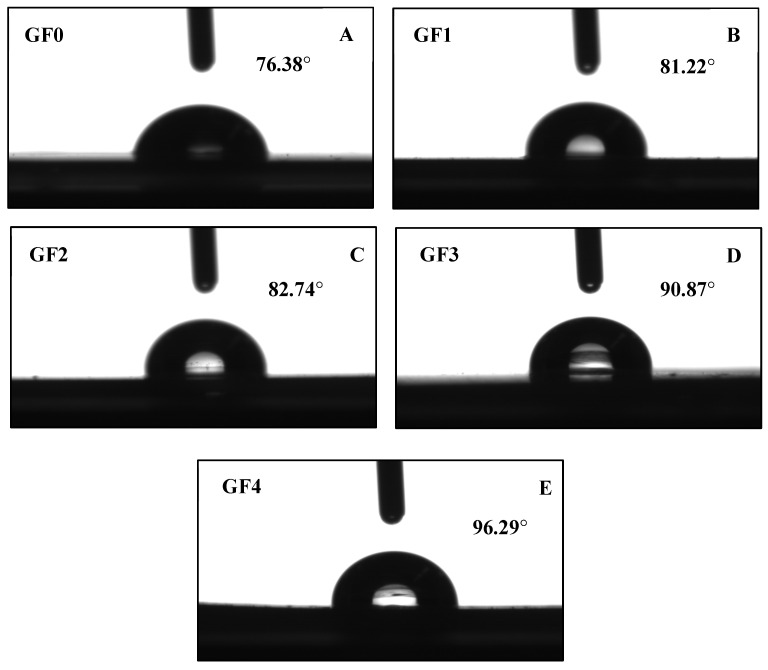
Water contact angle of gelatin capsule waste films incorporated with different levels of yellow peacock flower extract: (**A**) GF0, (**B**) GF1, (**C**) GF2, (**D**) GF3, and (**E**) GF4. GF0 represents gelatin film without yellow peacock extract, while GF1–GF4 represent films containing 0.25%, 0.5%, 1%, and 2% extract, respectively.

The hydrophobicity of gelatin capsule waste films containing different concentrations of YPE was assessed by water contact angle (WCA, θ_a(w)_) measurements, as shown in Figure 3 and Table 4. The control film (GF0) exhibited a WCA of 76.38°, whereas the film with the highest extract concentration reached 96.29°, indicating a significant increase in surface hydrophobicity (*p* < 0.05). This enhancement may be attributed to the uniform distribution of YPE within the gelatin matrix, where phenolic compounds engage in hydrogen bonding and hydrophobic interactions with gelatin chains. Such interactions likely reduce the number of exposed hydrophilic –OH groups, resulting in a more hydrophobic film surface [56]. The observed increase in WCA is consistent with the simultaneous decrease in water vapor permeability (WVP), confirming that YPE contributes to improved moisture resistance through structural modification of the protein network. Similar findings were reported by Song et al. [57], who observed increased WCA values in gelatin/agar films enriched with cranberry extract. Overall, the incorporation of YPE effectively improves the hydrophobic character of gelatin capsule waste films, reinforcing their potential for application in moisture-sensitive food packaging systems.

### 3.12. FTIR Spectroscopy

**Figure 4:**
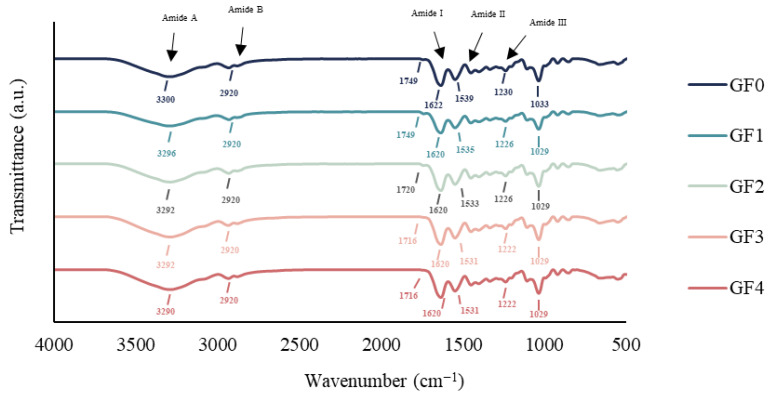
FTIR spectra of gelatin capsule waste films incorporated with different levels of yellow peacock extract. GF0: gelatin film without yellow peacock extract; GF1–GF4: gelatin films containing 0.25%, 0.5%, 1%, and 2% yellow peacock extract, respectively.

The FTIR spectra of gelatin capsule waste films incorporated with different concentrations of YPE are shown in Figure 4. All films exhibited a broad absorption band around 3300 cm^−1^ (Amide A), associated with inter- and intramolecular hydrogen bonding as well as O–H and N–H stretching vibrations [21]. A slight shift in this peak to a lower wavenumber (3290 cm^−1^) was observed in the film containing 2% YPE (GF4), indicating possible hydrogen bonding interactions between hydroxyl groups of polyphenols in the extract and the protein matrix [58]. A band near 2920 cm^−1^ (Amide B) was also detected in all samples, corresponding to asymmetric and symmetric stretching of methylene (C–H) groups, commonly associated with lipid components [14]. Additionally, absorption bands in the range of 1749–1700 cm^−1^ were observed, attributable to C=O ester stretching, likely derived from free fatty acids present in the gelatin capsule waste [59]. All films displayed characteristic gelatin peaks at 1622 cm^−1^, 1539 cm^−1^, and 1230 cm^−1^, corresponding to the Amide I (C=O stretching), Amide II (N–H bending and C–N stretching), and Amide III (C–N and N–H stretching) bands, respectively, confirming the protein backbone structure of gelatin [60]. A band at 1033–1029 cm^−1^ was also detected, attributed to –OH stretching of glycerol used as a plasticizer [61]. These results are consistent with those reported by Yarahmadi et al. [62], who observed similar spectral features in gelatin/chitosan films containing *Myrtus communis* L. extract. Overall, the spectral shifts, particularly in the Amide A region, suggest that YPE incorporation alters molecular interactions within the film matrix, primarily through hydrogen bonding between gelatin chains and phenolic compounds.

### 3.13. Thermogravimetric Analysis (TGA)

**Figure 5:**
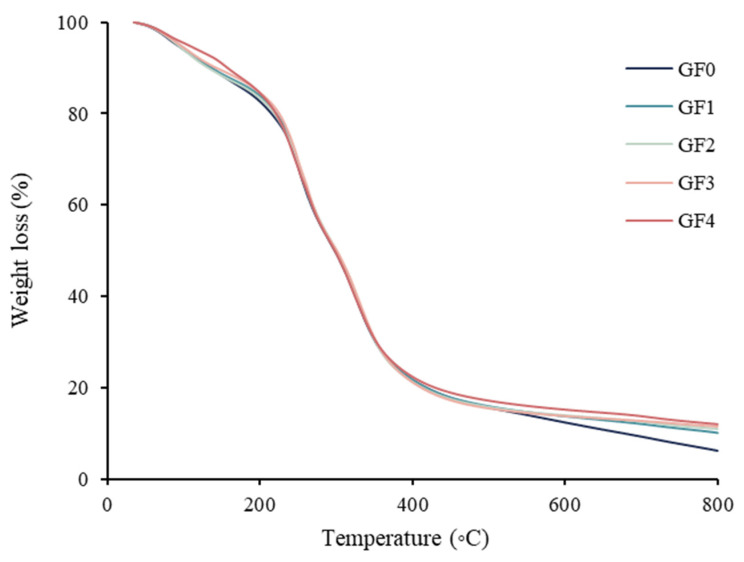
Thermogravimetric curves showing thermal degradation as weight loss (%) of gelatin capsule waste films incorporated with different levels of yellow peacock extract. GF0: gelatin film without yellow peacock extract; GF1–GF4: gelatin films containing 0.25%, 0.5%, 1%, and 2% yellow peacock extract, respectively.

The thermal degradation behavior of gelatin capsule waste films incorporated with different concentrations of YPE is shown in Figure 5. All films displayed three major weight-loss stages. The first degradation stage (Td_1_), occurring around 90 °C, corresponded to the evaporation of moisture, residual solvents, and other low-molecular-weight compounds present in the film matrix [63]. The second stage (Td_2_), observed near 236 °C, was attributed to the thermal degradation of glycerol, which acts as a plasticizer in the film formulation [64]. The final decomposition step (Td_3_), detected at approximately 330 °C, was associated with the breakdown of gelatin and other polymeric components [65]. Films containing YPE exhibited higher onset degradation temperatures and greater thermal stability compared to the control sample. This enhancement is likely due to the interaction between phenolic aromatic rings and the polymer chains, which may strengthen the structural network and limit thermal decomposition [66]. These observations are consistent with the findings of de Almeida Soares and de Aquino Santana [67], who reported improved thermal resistance in chitosan films incorporated with *Punica granatum* peel extract. Overall, the addition of YPE significantly modified the thermal degradation profile of the films, demonstrating its potential to enhance the stability of biodegradable gelatin-based materials.

### 3.14. Differential Scanning Calorimetry (DSC)

**Figure 6:**
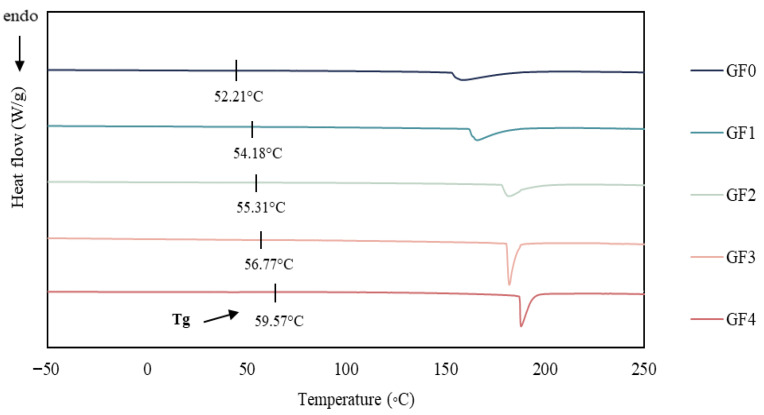
DSC thermograms of gelatin capsule waste films incorporated with different levels of yellow peacock extract. GF0: gelatin film without yellow peacock extract; GF1–GF4: gelatin films containing 0.25%, 0.5%, 1%, and 2% yellow peacock extract, respectively.

The DSC thermograms of gelatin capsule waste films (GCWF) containing different concentrations of YPE are shown in Figure 6. All samples exhibited endothermic transitions, indicating thermal events occurring within the polymer matrix. The control film (G0) displayed a lower glass transition temperature (Tg) compared to the YPE-incorporated films, suggesting that the addition of YPE improved the thermal stability of the gelatin network [67]. The highest Tg value was recorded for the film containing 2% YPE (GF4), implying that increasing extract concentration strengthened the interactions between gelatin chains and the phenolic constituents of YPE [41]. Endothermic peaks observed between 150 and 200 °C were attributed to crystallite melting and the release of bound water. While the control film (G0) exhibited a distinct peak at ~160 °C, YPE-enriched films showed broader peaks shifted toward higher temperatures. This shift reflects enhanced molecular rigidity and possible crosslinking promoted by phenolic–protein interactions, which increase the energy barrier for molecular motion and consequently elevate Tg. Comparable behavior was reported by Soltanzadeh et al. [68] in gelatin films containing pomegranate peel extract. Therefore, the incorporation of YPE markedly improved the thermal characteristics of gelatin capsule waste film, as demonstrated by increased Tg values and broadened endothermic transitions, confirming the role of phenolic compounds in reinforcing the film structure.

### 3.15. Antioxidant Activities of Film

**Figure 7:**
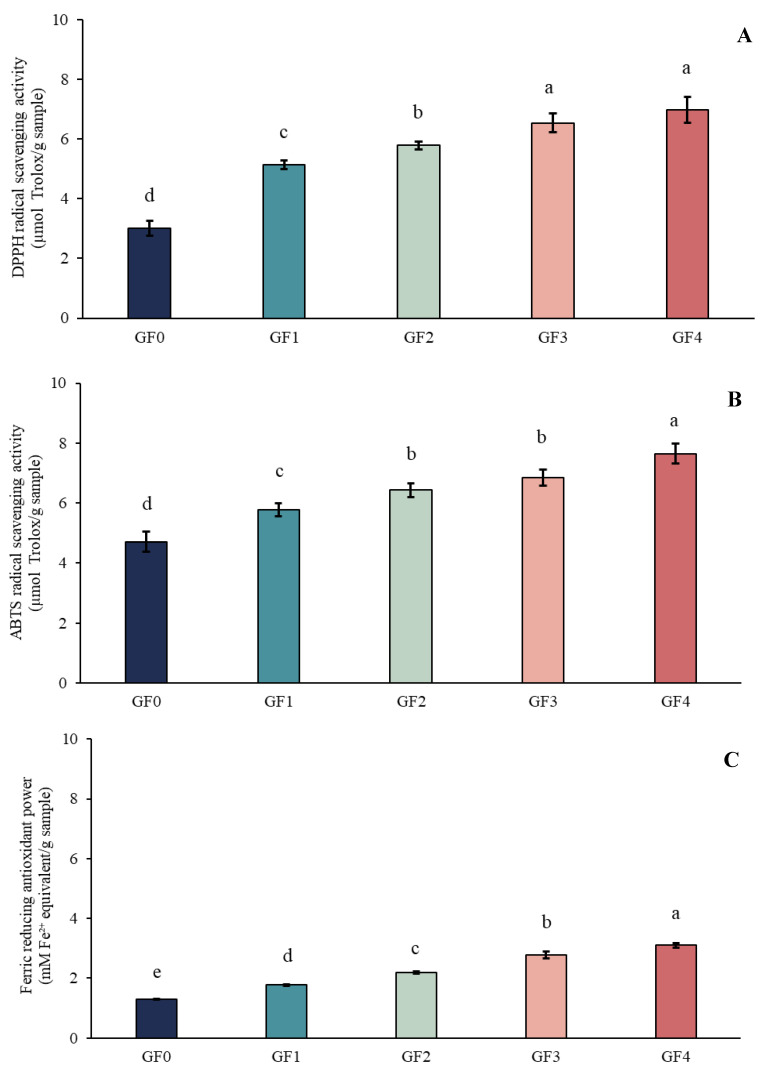
Antioxidant activities of gelatin capsule waste films incorporated with different levels of yellow peacock extract: (**A**) DPPH-RSA, (**B**) ABTS-RSA, and (**C**) FRAP. GF0: gelatin film without yellow peacock extract; GF1–GF4: gelatin films containing 0.25%, 0.5%, 1%, and 2% yellow peacock extract, respectively. The letters on the bars indicate significant differences (*p* < 0.05).

The antioxidant activities (ABTS, DPPH and FRAP) of gelatin capsule waste films (GCWF) containing different levels of YPE are presented in Figure 7. The use of multiple antioxidant assays (DPPH, ABTS, and FRAP) was intended to provide a comprehensive evaluation of antioxidant capacity, as each method is based on different reaction mechanisms, including radical scavenging and reducing power. A clear dose-dependent trend was observed, wherein higher YPE concentrations resulted in greater antioxidant capacity. The consistent trends observed across all assays indicate the reliability of the antioxidant performance of the films. The control film (GF0) exhibited the lowest activity, which can be attributed to the limited radical-scavenging ability of gelatin itself, despite the presence of amino and hydroxyl functional groups [69]. In contrast, films enriched with YPE showed a marked increase in antioxidant activity, confirming the contribution of the extract to the radical-quenching properties of the films. This improvement is mainly associated with the phenolic hydroxyl groups present in YPE, which are known to donate hydrogen atoms or electrons and thereby neutralize free radicals [70]. Similar behavior was reported by Ma et al. [71], who demonstrated enhanced antioxidant activity in chitosan/PVA films upon increasing concentrations of *Ginkgo biloba* leaf extract. Therefore, the results confirm that YPE functions as an effective antioxidant additive, significantly enhancing the functional performance of GCWF through its phenolic-based radical scavenging mechanisms.

### 3.16. Biodegradability: Indoor Soil Degradation

**Figure 8:**
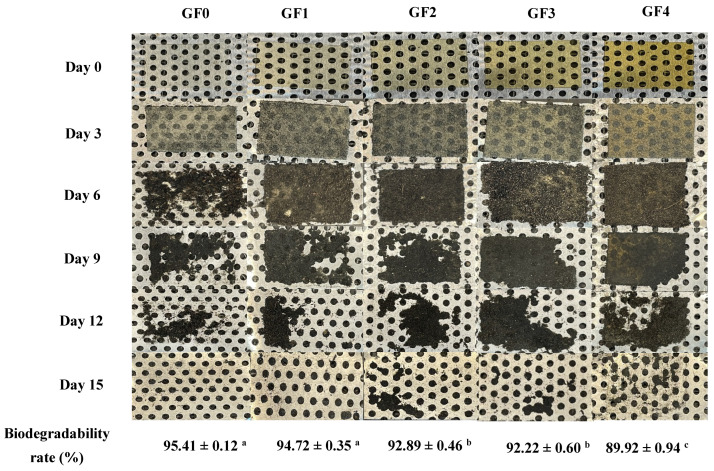
Biodegradability of gelatin capsule waste films incorporated with different levels of yellow peacock extract, and visual appearance of the films after burial in soil for 0–15 days. Different lowercase letters on the columns indicate significant differences (*p* < 0.05), and error bars represent ± standard deviation.

As an eco-friendly alternative to petroleum-derived plastics, the biodegradability of biopolymer-based films represents a key functional parameter of growing interest. Soil burial degradation is largely driven by microbial enzymes acting under aerobic and anaerobic conditions, which progressively depolymerize the material into simpler compounds that can be fully assimilated into the environment [72]. The biodegradation behavior of gelatin capsule waste films (GCWF) containing different concentrations of YPE is shown in Figure 8. All samples exhibited structural disintegration upon exposure to soil moisture, with complete degradation occurring within 15 days. However, the incorporation of YPE noticeably slowed the degradation rate of the films. This behavior is consistent with the solubility results, suggesting that YPE contributed to a more compact and less permeable film matrix. In addition, the phenolic constituents of YPE may impart antioxidative effects, thereby limiting microbial colonization and enzymatic attack, which could further delay biodegradation [73]. Similar trends were reported by El Mouzahim et al. [74], who observed reduced degradation rates in chitosan films containing *Ficus carica* leaf extract. Thus, GCWF demonstrated rapid and complete soil biodegradation without leaving harmful residues, confirming their environmental compatibility. The YPE-induced delay in degradation highlights a trade-off between functional enhancement and biodegradation rate, yet the films remain suitable for short-term packaging applications where controlled biodegradability is desirable.

### 3.17. Visual Appearance of Dried Shrimp During Storage

**Figure 9:**
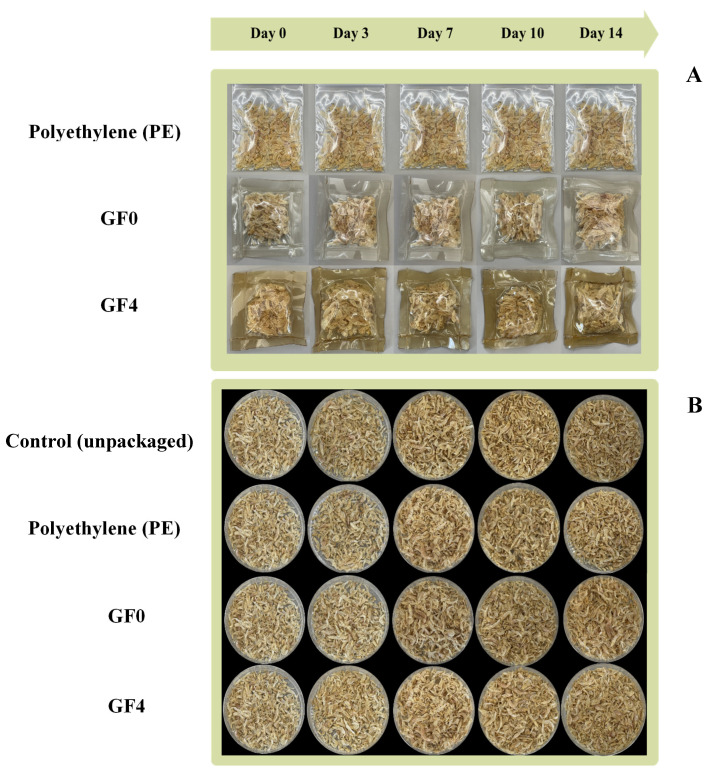
Visual appearance of gelatin films after sealing for 0–14 days (**A**), and visual appearance of dried shrimp after sealing for 0–14 days (**B**). GF0: gelatin film; GF4: gelatin film incorporated with 2% yellow peacock extract (YPE).

The visual appearance of dried shrimp packaged in gelatin films during storage is shown in Figure 9A,B. The dried shrimp packaged in polyethylene (positive control) exhibited negligible changes in color and appearance throughout the storage period compared to the other treatments. During the 14-day storage period, both the control samples (unpackaged dried shrimp) and dried shrimp packaged in GF0 exhibited a progressively darker, more yellowish coloration, accompanied by noticeable surface shrinkage and uneven drying. These visible changes are likely associated with moisture loss and increased oxidative reactions [75]. In contrast to the control samples, dried shrimp packaged in GF4 exhibited a more stable and uniform appearance throughout the storage period. Even at day 14, the dried shrimp packaged in GF4 retained a lighter color and exhibited less surface shrinkage, suggesting that the composite film effectively served as a protective barrier. The reduced discoloration may be attributed to the film’s ability to retard moisture loss, which is known to accelerate surface darkening during storage [76]. These observations further support the protective role of GF4 in retarding moisture evaporation and alleviating visual deterioration commonly associated with oxidative processes during storage.

### 3.18. Color Parameters of Dried Shrimp During Storage

**Figure 10:**
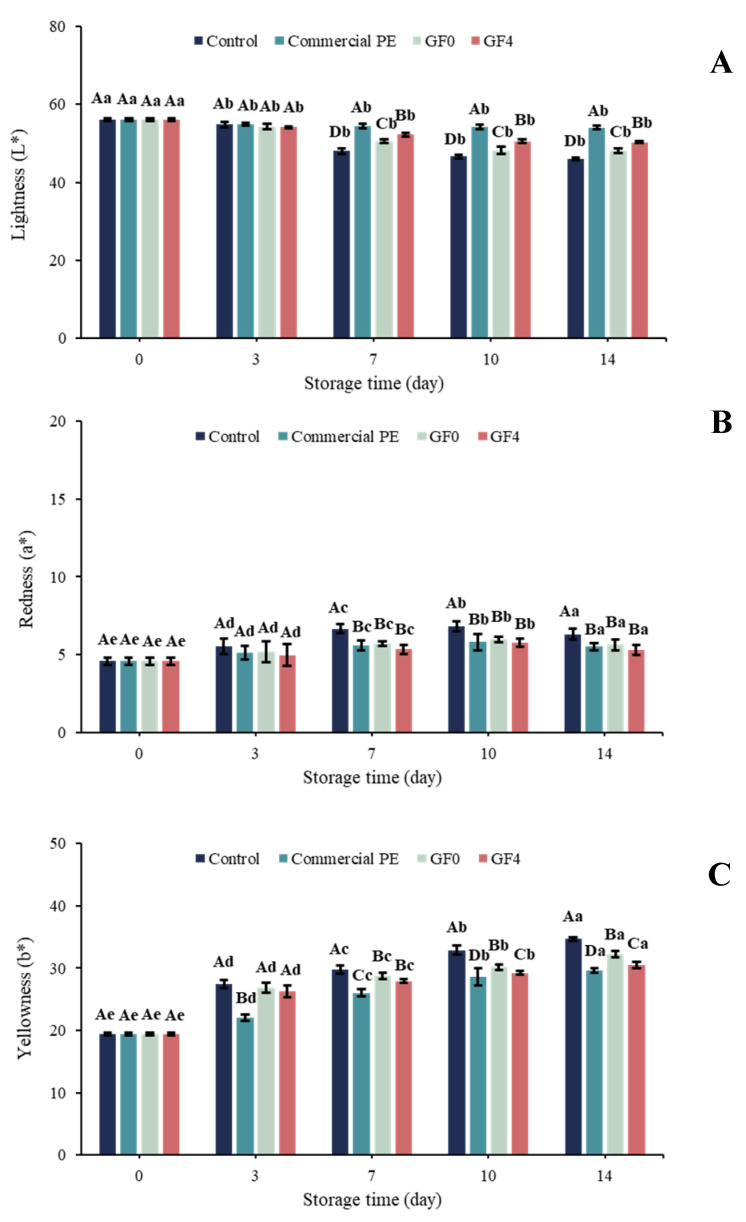
Changes in L* (**A**), a* (**B**), and b* (**C**) of dried shrimp during storage from 0 to 14 days. Uppercase letters indicate differences between treatments on the same day, whereas lowercase letters indicate differences between storage times. Means with the same letter are not significantly different (*p* > 0.05). GF0: gelatin film; GF4: gelatin film incorporated with 2% yellow peacock extract (YPE).

The color attributes of dried shrimp during storage are presented in Figure 10. At day 0, no significant differences (*p* > 0.05) were observed in lightness (L*), redness (a*), or yellowness (b*) between unpackaged and packaged dried shrimp. As storage progressed, L* values declined while a* and b* values increased in the unpackaged dried shrimp, indicating a transition in shrimp color from bright yellow to dark brown, which aligned with visual observations (Figure 9B). Negligible changes in color were observed in dried shrimp packaged in the GF4 film compared with those packaged in GF0 and the unpackaged dried shrimp. This may be attributed to the combined barrier properties of the gelatin matrix and the antioxidant activity of yellow peacock extract (YPE), which helped retard oxidative reactions in the shrimp [77]. The effectiveness of GF4 in reducing color deterioration of dried shrimp is consistent with recent developments in active gelatin-based packaging, such as gelatin films incorporated with shikonin, which have been reported to exhibit antioxidant activity when applied to shrimp packaging [78]. These findings further support that the incorporation of bioactive compounds into protein-based films can effectively preserve product quality by mitigating lipid oxidation during storage.

### 3.19. Moisture Content of Dried Shrimp During Storage

**Figure 11:**
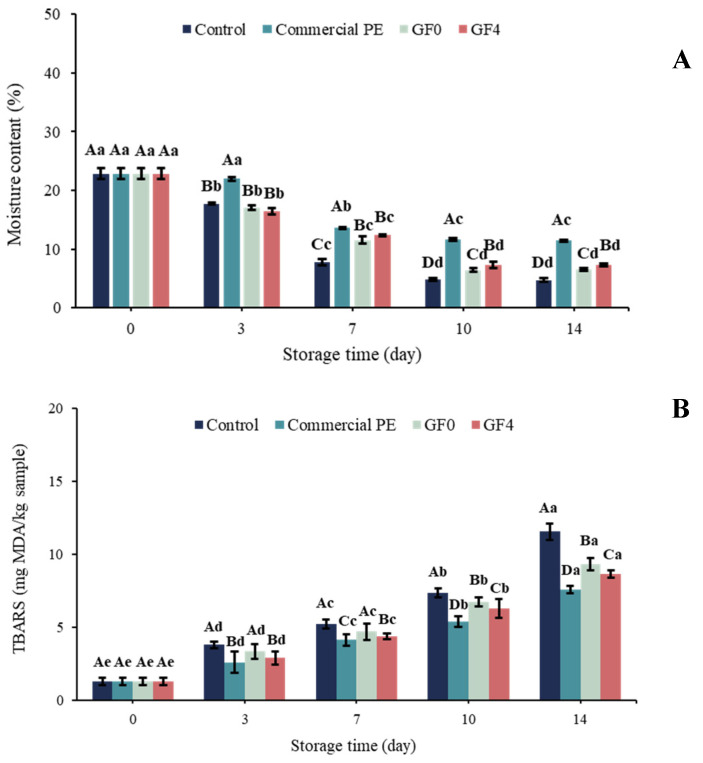
Moisture content (**A**) and TBARS values (**B**) of dried shrimp during storage from 0 to 14 days. Uppercase letters indicate differences between treatments on the same day, whereas lowercase letters indicate differences between storage times. Means with the same letter are not significantly different (*p* > 0.05). GF0: gelatin film; GF4: gelatin film incorporated with 2% yellow peacock extract (YPE).

Moisture content is a key quality indicator for dried shrimp, as it directly affects oxidation rate and overall shelf-life stability [75]. As shown in Figure 11A, dried shrimp packaged in polyethylene (positive control) exhibited higher moisture content throughout the storage period compared to the other treatments. This may be attributed to the excellent water vapor barrier properties of the plastic film, which effectively prevented moisture evaporation by restricting water vapor transmission through its polymeric matrix. This was evidenced by the condensation of water droplets on the inner surface of the packaging. After day 7, the unpackaged dried shrimp (control) consistently showed the lowest moisture content among all treatments throughout storage. This may be explained by unrestricted moisture evaporation in the absence of any protective barrier. Dried shrimp packaged in GF0 and GF4 exhibited slightly higher moisture content compared with the control samples. Although dried shrimp packaged in both GF0 and GF4 showed higher moisture content than the control samples, GF4 provided superior moisture retention compared with GF0, indicating a more effective barrier against water vapor transmission. This effect is likely due to the polyphenolic components of yellow peacock extract, which enhanced intermolecular interactions within the film matrix and formed a denser polymer network that restricted moisture migration. This observation aligns with Priyadharshee and Preetha [79], who found that gelatin films incorporating natural anthocyanins showed reduced moisture content and lower water vapor permeability due to increased structural compactness. Similarly, Taheri-Yeganeh et al. [80] reported that gelatin/pectin films enriched with pistachio peel anthocyanins improved moisture retention in shrimp, attributing this to decreased WVP and enhanced molecular bonding within the film matrix. Collectively, these findings reinforce the results of the present study, indicating that the GF4 film’s ability to retard moisture loss plays a crucial role in slowing lipid oxidation and preserving the overall quality of dried shrimp during storage.

### 3.20. Thio-Barbituric Acid Reactive Substances (TBARS)

The progression of lipid oxidation in dried shrimp during storage is illustrated in Figure 11B. The increase in TBARS values corresponds to partial dehydration and the oxidation of unsaturated fatty acids [81]. At day 0, no significant differences (*p* > 0.05) were detected among the unpackaged dried shrimp (control) and those packaged in GF0 and GF4. As storage time increased, TBARS values gradually increased across all treatments, indicating the accumulation of malondialdehyde (MDA), a secondary lipid oxidation product. Throughout storage, the control samples (unpackaged dried shrimp) showed the highest TBARS values (*p* < 0.05), corresponding to their greater yellowness (b), whereas polyethylene-packaged samples (positive control) exhibited the lowest TBARS values. Compared with GF0, samples packaged in GF4 exhibited slightly lower TBARS values, likely due to the antioxidant activity of yellow peacock extract (YPE), which contains polyphenols that scavenge free radicals and inhibit lipid peroxidation [82]. Similar trends were reported by Charoenphun et al. [83], who demonstrated that longkong pericarp extract helped maintain shrimp quality during storage. Overall, the reduced TBARS values in the GF4 treatment confirm that incorporating YPE into gelatin films provides protective effects against oxidative deterioration, thereby enhancing quality preservation and extending the shelf-life of dried shrimp.

## 4. Conclusions

This study demonstrates the effective valorization of gelatin capsule waste (GCW) as a biodegradable film matrix incorporated with yellow peacock extract (YPE) as a natural active agent. The YPE was rich in phenolic compounds, particularly flavonoid aglycones and their glycosylated derivatives, which exhibited strong antioxidant activity as confirmed by DPPH, ABTS, and FRAP assays. The incorporation of YPE significantly enhanced the antioxidant activity of the films, along with improved barrier and mechanical properties. All films exhibited rapid biodegradability, achieving complete degradation within 15 days. Among the formulations, the film containing 2% YPE (GF4) showed the most balanced physicochemical and functional performance. When applied to dried shrimp packaging, GF4 effectively reduced moisture loss and lipid oxidation during storage, resulting in improved product stability compared with unpackaged samples and performance comparable to polyethylene packaging. Overall, GCW–YPE films represent a promising biodegradable active packaging alternative capable of extending the shelf life of dried seafood while supporting circular economy and sustainability goals.

## Figures and Tables

**Table 1:** Total Phenolic content and antioxidant activities of the yellow peacock extract with different amplitude and extraction time.

Samples	TPC (mg GAE/g Dry Extract)	DPPH-RSA (mg TE/g Dry Extract)	ABTS-RSA (mg TE/g Dry Extract)	FRAP (mg TE/g Dry Extract)
E0	88.14 ± 0.01 ^e^	2.87 ± 0.25 ^f^	4.97 ± 0.08 ^f^	3.47 ± 0.36 ^f^
E1	98.44 ± 0.05 ^d^	3.17 ± 0.30 ^f^	5.51 ± 0.04 ^e^	3.86 ± 0.09 ^ef^
E2	101.94 ± 0.02 ^c^	3.73 ± 0.19 ^e^	5.66 ± 0.06 ^d^	4.03 ± 0.08 ^bcd^
E3	102.82 ± 0.15 ^c^	4.63 ± 0.36 ^cd^	5.91 ± 0.06 ^c^	4.33 ± 0.08 ^bcd^
E4	104.84 ± 0.06 ^bc^	4.92 ± 0.21 ^c^	5.98 ± 0.12 ^c^	4.49 ± 0.21 ^bc^
E5	113.77 ± 0.03 ^b^	6.70 ± 0.14 ^b^	6.93 ± 0.09 ^b^	4.61 ± 0.41 ^b^
E6	129.34 ± 0.02 ^a^	7.63 ± 0.20 ^a^	8.11 ± 0.01 ^a^	5.82 ± 0.22 ^a^

Values are expressed as mean ± standard deviation (SD) (n = 3). Different lowercase superscript letters indicate significant differences (*p* < 0.05). Treatments: E0 = maceration; E1–E3 = UAE at 20% amplitude (15, 30, and 45 min); E4–E6 = UAE at 40% amplitude (15, 30, and 45 min).

**Table 2:** Phenolic compounds identified in the yellow peacock flower extract.

Rt (min)	Compound	Molecular Formula	Molecular Weight (Da)	QTOF-ESI-MS (Negative Ion Mode)	
				Precursor Ion (*m*/*z*)	MS/MS Fragments (*m*/*z*)
**Phenolic acids**					
0.4623	Quinic acid	C_8_H_16_OS_2_	192.17	191.0566	85.0285/93.0343/191.0565
0.5472	Gallic acid	C_7_H_6_O_5_	170.12	169.0168	79.0192/124.0171
0.7771	Calceorioside B	C_12_H_26_N_6_O_10_S_2_	478.45	477.1414	477.1414
**Flavonoid**					
1.5024	Bavachinin A ^b^	C_13_H_30_N_4_O_6_	338.4	337.208	337.2065/96.9600
2.9909	Luteoloside	C_17_H_36_O_5_S_4_	448.37	447.1369	447.1362
**Glycoside**					
0.5849	Resibufogenin +HCOOH	C_22_H_22_N_8_O_2_	430.47	429.1786	429.1756/169.0147
**Carboxylic acids**					
0.4725	Amber Acid ^a^	C_4_H_6_O_4_	118.09	117.0216	72.0296/55.0199
0.5067	L-Malic acid	C_4_H_6_O_5_	134.09	133.0163	71.0139/72.9934/115.0034
0.5755	Citric acid	C_7_H_4_N_4_O_3_	192.12	191.0217	87.0085/111.0085/85.0292
**Saccharide**					
0.4534	D-(+)-Mannose	C_6_H_12_O_6_	180.16	179.0589	59.0136/71.0139/72.9932

^a^ Compound detected only in YPE control extract (E0); ^b^ Compound detected only in YPE obtained under the optimal UAE condition (E6).

**Table 3:** Color and Transparency value of the gelatin capsule waste incorporated with different yellow peacock extract levels (0–2%).

Samples	L*	a*	b*	∆E	Transparency Value
GF0	88.68 ± 0.24 ^a^	−1.93 ± 0.14 ^c^	14.57 ± 0.87 ^e^	-	0.2814 ± 0.0107 ^b^
GF1	86.77 ± 0.56 ^b^	−1.76 ± 0.03 ^c^	21.32 ± 0.87 ^d^	8.01 ± 0.90 ^d^	0.2834 ± 0.0019 ^b^
GF2	85.28 ± 0.67 ^c^	−1.55 ± 0.17 ^b^	28.86 ± 0.76 ^c^	15.67 ± 0.80 ^c^	0.2843 ± 0.0150 ^b^
GF3	84.85 ± 0.64 ^c^	−1.47 ± 0.19 ^b^	31.99 ± 0.68 ^b^	18.81 ± 0.73 ^b^	0.3064 ± 0.0386 ^b^
GF4	77.74 ± 0.37 ^d^	3.49 ± 0.33 ^a^	52.39 ± 0.72 ^a^	40.69 ± 0.75 ^a^	0.3939 ± 0.0422 ^a^

Values are expressed as mean ± standard deviation (n = 10). Different lowercase superscript letters in the same column indicate significant differences (*p* < 0.05). GF0: gelatin film without yellow peacock extract; GF1–GF4: gelatin films containing 0.25%, 0.5%, 1%, and 2% yellow peacock extract, respectively.

**Table 4:** Thickness, tensile strength, elongation at break, moisture content, solubility, water vapor permeability, and water contact angle of the gelatin capsule waste films incorporated with different yellow peacock extract levels (0–2%).

Samples	Thickness (mm)	Tensile Strength (MPa)	Elongation at Break (%)	Moisture Content (%)	Solubility (%)	WVP (×10^−11^ g·m·m^−2^·s^−1^·Pa^−1^)	Water Contact Angle θ_a_(w) (°)
GF0	0.2613 ± 0.0228 ^d^	2.25 ± 0.15 ^c^	36.67 ± 3.14 ^a^	19.58 ± 0.37 ^a^	88.78 ± 0.67 ^a^	1.76 ± 0.05 ^a^	76.38 ± 0.95 ^e^
GF1	0.3018 ± 0.0411 ^c^	2.31 ± 0.13 ^c^	26.33 ± 1.89 ^b^	19.54 ± 0.40 ^a^	82.16 ± 0.95 ^b^	1.62 ± 0.03 ^b^	81.22 ± 0.31 ^d^
GF2	0.3196 ± 0.0242 ^bc^	2.39 ± 0.15 ^bc^	18.67 ± 2.33 ^c^	17.73 ± 0.62 ^b^	67.69 ± 0.51 ^c^	1.42 ± 0.01 ^c^	82.74 ± 0.01 ^c^
GF3	0.3387 ± 0.0179 ^ab^	2.53 ± 0.33 ^b^	17.33 ± 2.11 ^d^	17.39 ± 0.71 ^b^	60.74 ± 0.59 ^d^	1.36 ± 0.04 ^c^	90.87 ± 0.04 ^b^
GF4	0.3472 ± 0.0213 ^a^	2.80 ± 0.21 ^a^	13.67 ± 2.46 ^e^	17.10 ± 0.28 ^b^	59.07 ± 0.42 ^e^	1.22 ± 0.02 ^d^	96.29 ± 0.02 ^a^

Values are expressed as mean ± standard deviation (n = 10). Different lowercase superscript letters in the same column indicate significant differences (*p* < 0.05). GF0: gelatin film without yellow peacock extract; GF1–GF4: gelatin films containing 0.25%, 0.5%, 1%, and 2% yellow peacock extract, respectively.

## Data Availability

The original contributions presented in this study are included in the article/Supplementary Material. Further inquiries can be directed to the corresponding author.

## Supplementary Materials

The following supporting information can be downloaded at: https://www.mdpi.com/article/10.3390/antiox15050576/s1, Table S1: LC–MS/MS-identified phenolic compounds in yellow peacock leaf extract obtained by maceration (control sample, E0).
