# Supplementary material for: Ultrasound-Assisted Extraction of Yellow Peacock Flower (Caesalpinia pulcherrima) and Its Application in Gelatin Capsule Waste-Based Active Packaging Films for Dried Shrimp Preservation
Source: Antioxidants (Basel). 2026 May 2;15(5):576. doi: 10.3390/antiox15050576 (PMC13203943; doi:10.3390/antiox15050576)
Supplement: Supplementary file 1 [file antioxidants-15-00576-s001.zip › antioxidants-4190147-supplementary.pdf]

Article

Ultrasound-Assisted Extraction of Yellow Peacock Flower Extract and Its Application in Gelatin Capsule Waste-Based Active Packaging Films for Dried Shrimp Preservation

Pudthaya Kumnerdsiri[1], Khanittha Chinarak[1], Lalitphan Kitsanayanyong[1], Anurak Uchuwittayakul[2], Wanchat Sirisarn[3], Piyangkun Lueangjaroenkit[4], Pimonpan Kaewprachu[5], Jaksuma Pongsetkul[6], Samart Saiut[7], Saroat Rawdkuen[8], and Passakorn Kingwascharapong[1,*]

1 Department of Fishery Products, Faculty of Fisheries, Kasetsart University, Bangkok 10900, Thailand; pudthaya.ku@ku.th; khanittha_noo@hotmail.com; lalitphan.ki@ku.th
2 Department of Aquaculture, Faculty of Fisheries, Kasetsart University, Bangkok 10900, Thailand; ffisarb@ku.ac.th
3 Department of Microbiology, Faculty of Medicine, Kasetsart University, Bangkok 10900, Thailand; wanchat.s@ku.th
4 Department of Microbiology, Faculty of Science, Kasetsart University, Bangkok 10900, Thailand; Piyangkun.lu@ku.th
5 Faculty of Agro-Industry, Chiang Mai University, Samut Sakhon, 74000, Thailand; Pimonpan.k@cmu.ac.th
6 School of Animal Technology and Innovation, Institute of Agricultural Technology, Suranaree University of Technology, Nakhon Ratchasima 30000, Thailand; jaksuma@sut.ac.th
7 Department of Food Science, Faculty of Science, Burapha University, Chonburi. 20131, Thailand; samarts@go.buu.ac.th
8 Unit of Innovative Food Packaging and Biomaterials, School of Agro-Industry, Mae Fah Luang University, Chiang Rai 57100, Thailand; saroat@mfu.ac.th
* Correspondence: passakorn.ki@ku.th

Supplementary Table S1. LC–MS/MS-identified phenolic compounds in yellow peacock leaf extract obtained by maceration (control sample, E0).

RT (min)	Compounds	Precursor ion (m/z)	Molecular formula
0.09	2-(Decylsufinyl)ethanol	233.1580	$C_{12}H_{26}O_2S$
0.17	bis[2-hydroxyethyl-[2-(2-hydroxyethylamino)ethyl]amino] carbonate	353.2041	$C_{13}H_{30}N_4O_7$
0.28	N-Benzyl-5-phenyl-1H-pyrazole-3-carboxamide	276.1145	$C_{17}H_{15}N_3O$
0.35	2,3-Dimethyl-5-(2-thienyl)-1,3,4-thiadiazole	272.9586	$C_{15}H_2N_2S_2$
0.37	2,3,5,6-Tetrachlorobenzothiophene-1,1-dioxide	440.8849	$C_{19}H_6O_3S_5$
0.37	1,3,5-Triazine-2,4,6-trithiol-1,3,5-triazine-2,4,6-trithiol	242.9433	$CH_4N_6O_3S_3$
0.40	(Coronen-1-yl)methylidene]hydrazine	341.1081	$C_{25}H_{14}N_2$
0.45	Odoracin	533.1750	$C_{37}H_{26}O_4$
0.55	2-[[5-(4-phenylphenyl)-[1,3]thiazolo[5,4-d][1,3]oxazol-2-yl]methylidene]cyclopenta[b]naphthalene-1,3-dione	483.0810	$C_{30}H_{16}N_2O_3S$
0.59	Methyl 2-methyl-2-propanyl 2,2′-sulfonyldiacetate	251.0595	$C_9H_{16}O_6S$
0.78	3-sulfooxybutanoic acid	182.9967	$C_4H_8O_6S$
0.81	3-Hydroxy-6-methyl-8-nitro-2-(1,2,3,4-tetrahydroxybutyl)-2,3-dihydroimidazo[1,2-c]pyrimidin-5,7(1H,6H)-dion	347.0852	$C_{11}H_{16}N_4O_9$
0.87	1-oxa-6,9,12-trithiacyclohexadecane	279.0916	$C_{12}H_{24}OS_3$
1.23	5-(2,5-diazabicyclo[2.2.1]heptan-2-yl)-3-methyl-1,2,4-thiadiazole	195.0709	$C_8H_{12}N_4S$
1.50	1,4-Bis(morpholinoacetyl)piperazine	339.2035	$C_{16}H_{28}N_4O_4$
1.52	1-cyclohexyl-3-[5-(1H-imidazol-5-yl)pentyl]thiourea	293.1805	$C_{15}H_{26}N_4S$
1.76	Squairc acid	112.9879	$C_4H_2O_4$
1.94	3-amino-1-(butylsulfamoylamino)-6-methylheptan-2-ol	294.1858	$C_{12}H_{29}N_3O_3S$
2.00	N-[3-(trinitromethyl)-1H-1,2,4-triazol-5-yl]nitramide	276.9921	$C_3H_2N_8O_8$
2.22	N-[(3-Carboxybenzyl)sulfonyl]-L-isoleucyl-N~1~-(4-carbamimidoylbenzyl)-L-glutamamide	587.2293	$C_{27}H_{36}N_6O_7S$
2.80	6-dodecylsulfanyl-2-(4-heptoxyphenyl)-1,3-benzothiazole;methane	540.3341	$C_{33}H_{51}NOS_2$
3.32	(2S,3S,4R)-N-ethyl-3,4-dihydroxy-5-[6-[6-[(4-nitro-2,1,3-benzoxadiazol-7-yl)amino]hexylamino]purin-9-yl]oxolane-2-carboxamide	569.2226	$C_{24}H_{30}N_{10}O_7$
3.96	N,N-Dicyclohexyl-2-(2-methylphenyl)-4-quinolinecarboxamide	425.2596	$C_{29}H_{34}N_2O$
4.29	Coelenteramine	276.1142	$C_{17}H_{15}N_3O$
4.32	3-amino-1-(butylsulfamoylamino)-6-methylheptan-2-ol	294.1857	$C_{12}H_{29}N_3O_3S$
4.44	Infigratinib	571.2346	$C_{20}H_{44}N_8O_3S_4$
5.03	4-Imidazolidinesulfonic acid, 1-(2-methylpropyl)-, (3R,5S)-1-methyl-5-[[[[1-(2-methylpropyl)-4-imidazolidinyl]sulfonyl]amino]methyl]-3-pyrrolidinyl ester	509.2585	$C_{20}H_{42}N_6O_5S_2$

5.42	[3-[4-methoxy-3-(3-methoxypropoxy)benzoyl]-4-methyl-1-(5-oxo-4-propan-2-yloxolan-2-yl)pentyl] 4-methylbenzenesulfonate	603.2632	$C_{32}H_{44}O_9S$
9.46	ethyl 2-[2-[[9-[2-[(2-ethoxy-2-oxoethyl)carbamothioylamino]ethylcarbamoyl]-4-(4-methylphenyl)-3,5-dioxatricyclo[5.2.1.02,6]decane-8-carbonyl]amino]ethylcarbamothioylamino]acetate	691.2588	$C_{31}H_{44}N_6O_8S_2$
12.29	(2S)-6-amino-2-(hydroxyamino)hexanoic acid;(2S)-2-amino-6-(methylamino)hexanoic acid	321.2141	$C_{13}H_{30}N_4O_5$
